# Open-source lab hardware: Driver and temperature controller for high compliance voltage, fiber-coupled butterfly lasers

**DOI:** 10.1016/j.ohx.2021.e00240

**Published:** 2021-10-16

**Authors:** Florian Kehl, Vlad F. Cretu, Peter A. Willis

**Affiliations:** a NASA Jet Propulsion Laboratory, California Institute of Technology, Pasadena, CA 91109, USA; b Innovation Cluster Space and Aviation (UZH Space Hub), Air Force Center, University of Zurich, 8600 Dübendorf, Switzerland; c Institute of Anatomy, Faculty of Medicine, University of Zurich, 8057 Zurich, Switzerland; d Institute of Medical Engineering, Space Biology Group, Lucerne University of Applied Sciences and Arts, 6052 Hergiswil, Switzerland

**Keywords:** Laser control, Optical sensing, Butterfly laser, High compliance voltage

## Abstract

This article describes the development of a compact, relatively low-cost, high compliance voltage laser driver that can provide the constant optical laser output required for a range of applications. The system contains an integrated, high-precision temperature controller that can be implemented with butterfly-style lasers containing an internal thermoelectric cooler. The laser parameters can be controlled manually or via an onboard microcontroller. Additionally, an adjustable over-current protection circuit safeguards the laser diode from potential damage.

## Graphical abstract

**Figure:**
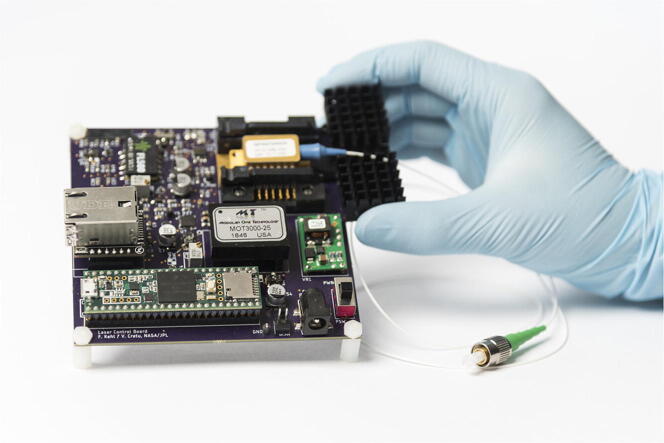

## Specifications Table

**Table:** 

**HardwareName**	**Open-Source Butterfly Laser Driver**
Subject Area	• Electrical Engineering • Optics
Hardware Type	• Laser Driver
Open Source License	*CC BY 4.0*
Cost of Hardware	*$398.50 without network module,* $422.50 USD with network module*
Source File Repository	*https://doi.org/10.17605/OSF.IO/R65MQ*

*The cost information contained in this document is of a budgetary and planning nature and is intended for informational purposes only. It does not constitute a commitment on the part of JPL and/or Caltech.

## Hardware in Context

Since their invention over sixty years ago, lasers have become an essential tool for virtually all branches of science, technology, and engineering. The coherent light of laser sources has enabled novel techniques and discoveries in physics, chemistry, biology, medicine, environmental sciences, and remote sensing [1], [2], [3], [4], and also led to new processes and applications in manufacturing, communications, and consumer electronics [5], [6], [7]. The latter two fields were the primary drivers for the advancement, miniaturization, and mass production of solid-state lasers, which provided affordable coherent sources covering the infrared as well as the visible spectrum. While the availability of a broad spectrum of lasers opened up many new possibilities, research-grade laser controllers that provide a stable and reproducible optical output remain relatively expensive and bulky. Additionally, especially for short emission wavelengths of 405 or 488 nm, both attractive wavelengths for laser-induced fluorescence (LIF) experiments [8], the compliance, or forward-voltage, V_f_, of diode lasers can be higher than supported by many commercial laser controllers. If it is the reader’s intent to build a custom LIF system, the authors would like to refer to two other parts of the series “Open-Source Lab Hardware” [9], [10].

**Fig. 1:**
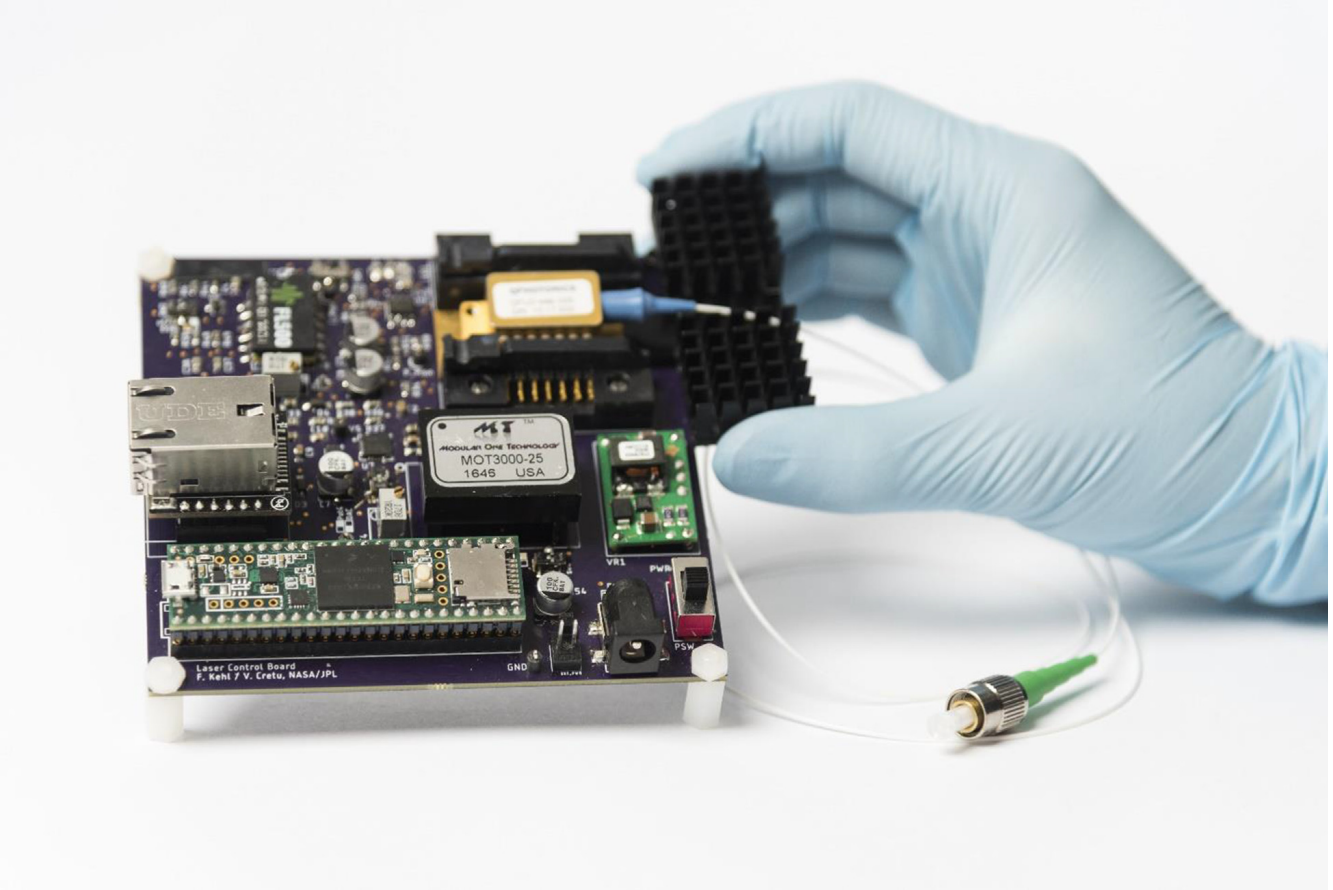
Photograph of the fully assembled laser driver board.

Here, we present a compact, relatively inexpensive high-V_f_ laser driver with an integrated, high-precision temperature controller for lasers with an internal thermoelectric cooler (TEC), equipped with a microcontroller for direct control via a computer (Fig. 1). The laser diode (LD) can be run in constant current (CC) mode with up to 250 mA of forward current (I_f_) and V_f_ of up to 8 V, or in constant power (CP) mode if the laser package contains an internal photodiode (PD). The board is designed for a fiber-coupled, 14-pin butterfly laser to facilitate the connection of the laser source to any auxiliary equipment. Power/current and temperature can either be set via potentiometers on the hardware itself or by serial commands via USB or Ethernet. This driver is primarily designed for continuous wave (CW) laser operation and provides telemetry regarding laser temperature as well as LD, PD, and TEC current. The board also has adjustable hardware over-current protection to safeguard the laser from inadvertently high currents. This hardware is intended as a low-cost, open-source alternative to existing tools, such as the LDTC0520 Laser Diode Driver from Team Wavelength (Wavelength Electronics, Inc., MT, USA), or the CLD1015 Compact LD and Temperature Controller with Mount from Thorlabs (Thorlabs, NJ, USA).

## Hardware Description

This laser driver board was custom-designed using Eagle PCB (Autodesk, CA, USA) as a 2-layer printed circuit board (PCB), which can be fabricated in any board house or a PCB milling machine. For the PCB presented in this work, we chose OSH Park (Oshpark LLC, OR, USA) as the manufacturer. The individual components are commercial off-the-shelf (COTS), as detailed in the bill of material (BOM), and are directly mounted to the PCB. For people with less soldering experience, there are also companies that offer turn-key solutions and a fully assembled board when provided layout and BOM.

The board is equipped with a Teensy 3.5 microcontroller to power a 14-pin butterfly laser via a commercial laser driver and TEC controller. Its main features are:

• Low-cost, open-source design
• Compatibility with lasers that exhibit a high compliance voltage (V_f_) of up to 8 V, and up to 250 mA forward current (I_f_)
• Operability in either constant current (CC) or constant power (CP) modes
• A high precision temperature controller
• An over-current protection circuit to avoid damage to the laser
• An onboard microcontroller which allows for remote control and measurement of laser current/power and temperature via serial commands or Ethernet

In the following, the hardware is described in more detail.

### Microcontroller

**Fig. 2:**
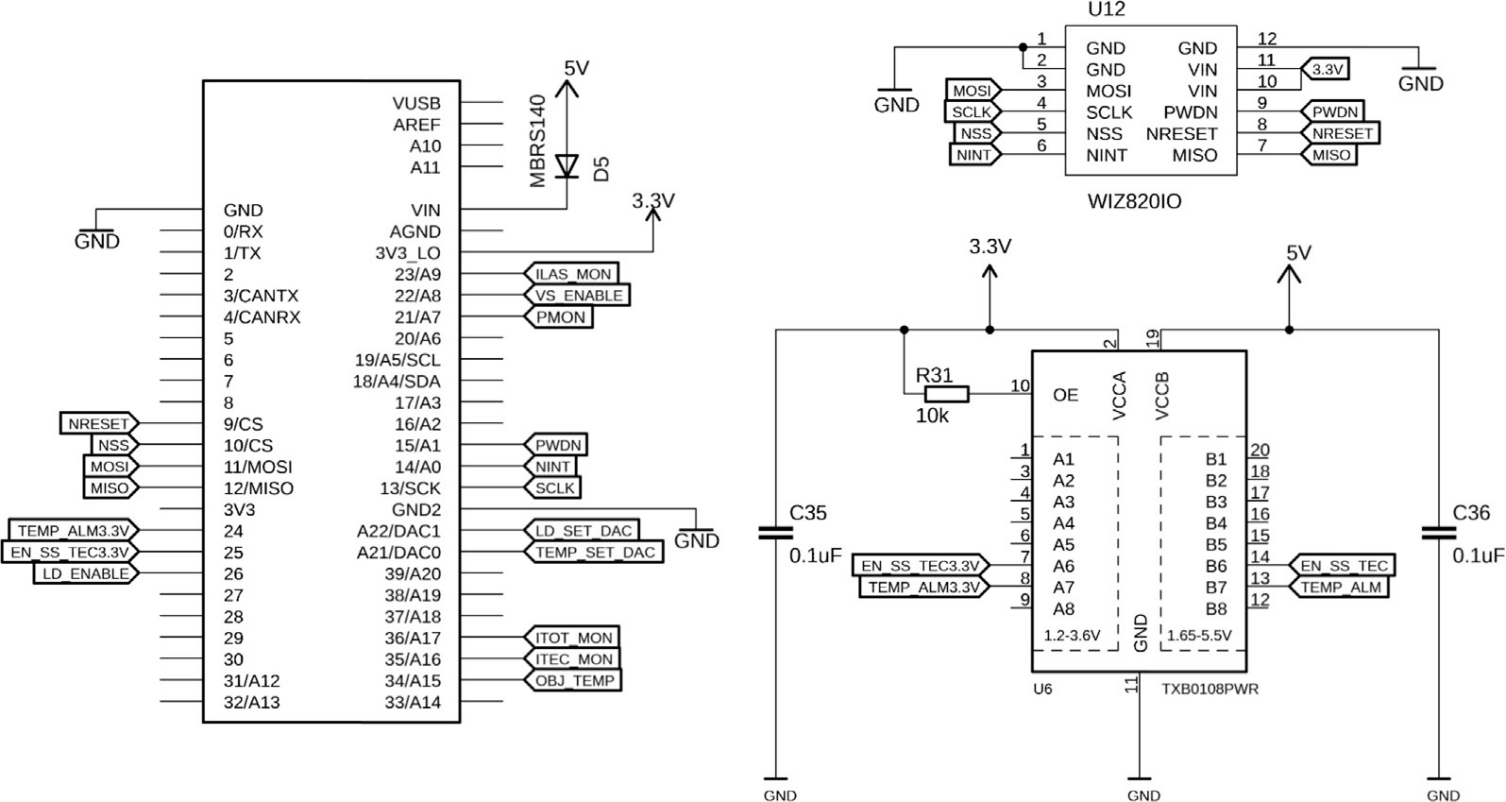
Pin layout of the Teensy 3.5 microcontroller, together with a 3.3 – 5 V level shifter (U6) for digital I/Os and the optional Ethernet module (U12).

At its core, the board is controlled by a Teensy 3.5 development board (PJRC, CT, USA), a USB-based microcontroller development system (Fig. 2). It features a 32-bit 120 MHz ARM Cortex-M4 processor with floating point unit, with 512 and 256 kB flash memory and RAM, respectively. With 58 digital I/O, 27 analog inputs at 13 Bits, two analog outputs at 12 Bits, 20 pulse-width-modulated outputs, various communication protocols/bus systems such as USB, Serial, I^2^C, SPI and CAN bus, and an SD card, it is well equipped to control and read from a multitude of drivers and integrated circuits. The board also hosts an Ethernet port, as this might be the preferred communication interface for certain applications. All programming is done via the USB port, and the platform is compatible with the Arduino software and its libraries. The source code for this project, including all the required libraries, is made available in the supplementary materials. The Teensy can be powered directly through the USB cable, but here we strongly recommend powering it via its VIN pin by applying 5 V, provided directly by the board. To avoid competition between the 3.3 V provided from the USB and the 5 V from the board, we recommend cutting the VUSB line, which is explained in *Section Build Instructions*.

### Voltage Converters and Current Measurement

**Fig. 3:**
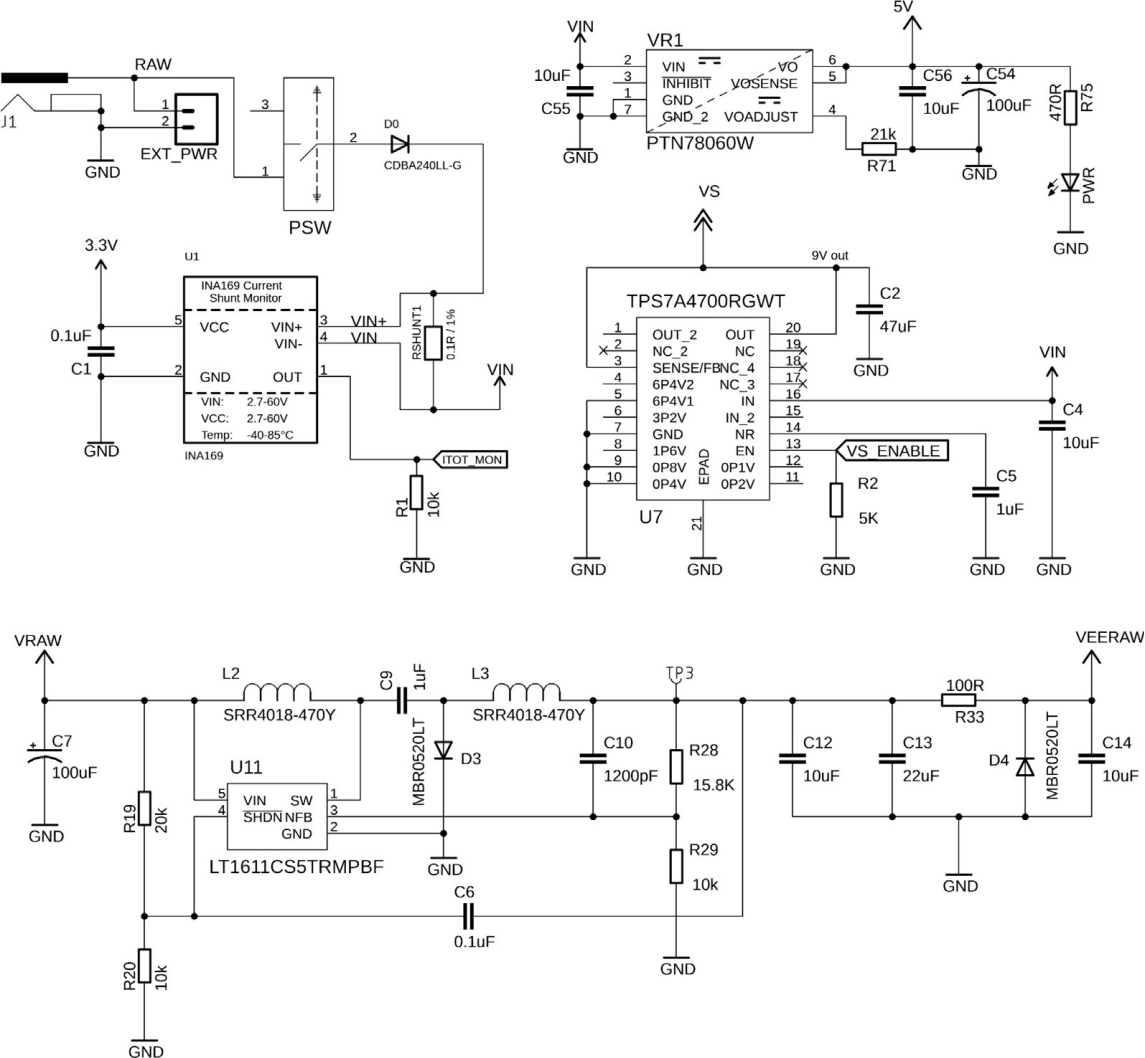
External 12 V power is supplied through J1 or alternatively the EXT_PWR pin header. The overall current, and hence power consumption of the board, can be measured by U1. VR1 provides the 5 V rail. U7 is an ultralow noise, low-dropout linear regulator which provides the necessary 9 V at VS required from the input 12 V. This voltage is necessary to power the laser controller as well as provide the voltage across the laser diode. Similarly, U11 is a low noise inverting regulator configured to output a small negative voltage at VEERAW. VEERAW, at −0.1 V, and VS provide the rails for various op-amps to control the laser diode.

The board is powered via a 5.5 × 2.1 mm barrel connector (J1) or a 2-pin header (EXT_PWR). We recommend applying 12 VDC at the voltage input (VIN), e.g., by connecting a wall adapter with sufficient power such as the Qualtek QFWB-65-12-US01, which can provide up to 5 A at 12 VDC. The input voltage is directly fed into a voltage regulator (VR1) to convert the 12 VDC input into 5 VDC (Fig. 3). The 3.3 VDC rail is provided by the Teensy's internal voltage regulator. The input voltage is supplied, via a series of low-pass filter LRC circuits (not shown in Fig. 3), to a low-dropout linear regulator (U7) and a low-noise, inverting switching regulator (U11) to provide the positive and negative rails to bias the laser diode. The power to the board can be cut by a sliding switch (PSW), its state indicated by PWR_LED. The board's overall current can be measured by an INA169 (U1) current monitor chip, which can provide useful telemetry about the system and overall current (and hence also power) consumption of the board and its components.

### Laser Mount

**Fig. 4:**
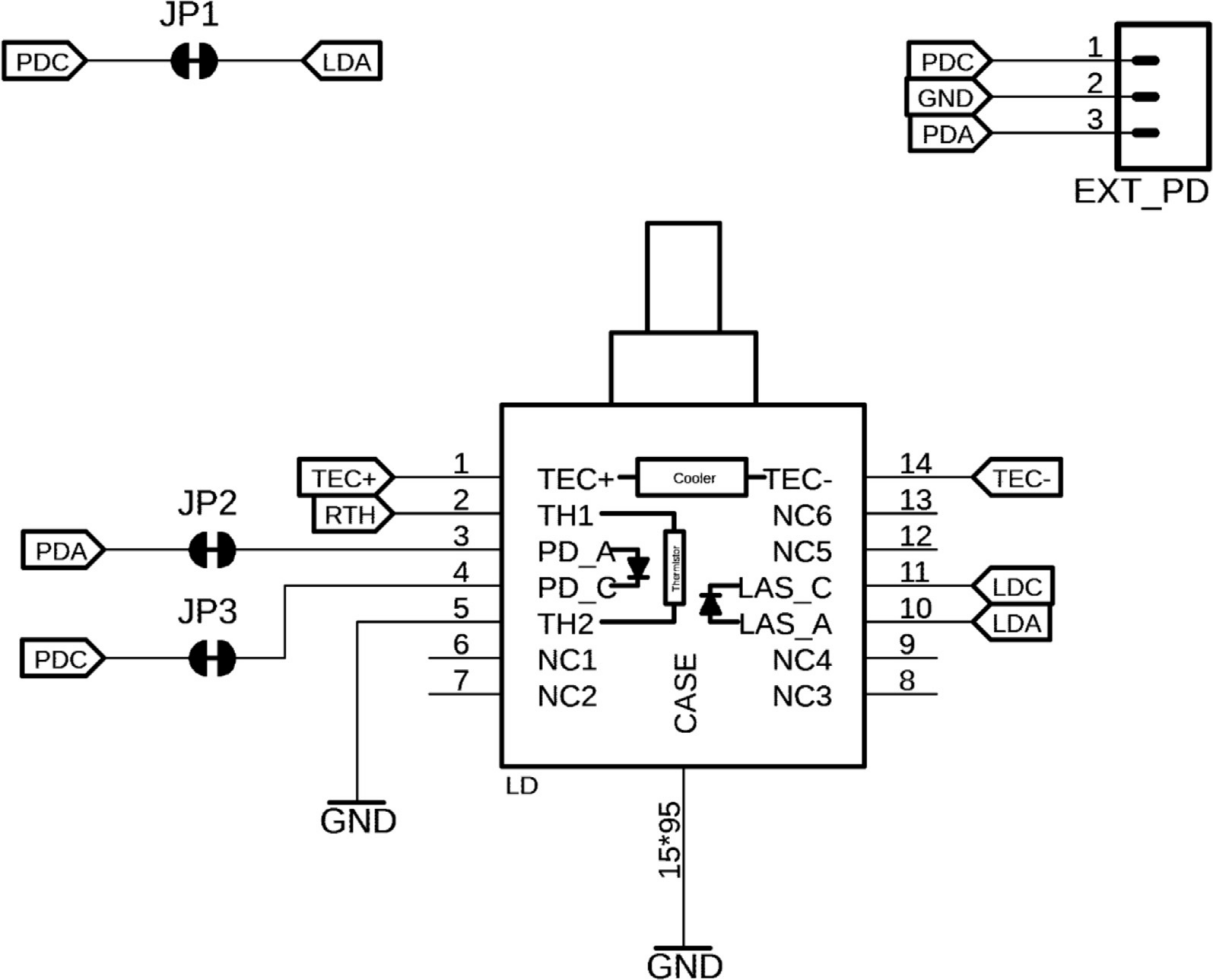
14-pin butterfly laser diode (LD) with its internal elements: laser diode, feedback photodiode, thermistor, and thermoelectric cooler. If an external photodiode is desired, jumpers JP 1–3 have to be cut. 1: TEC (+), 2: Thermistor, 3: PD Anode (+), 4: PD Cathode (−), 5: Thermistor, 6: Not Connected (NC), 7: NC, 8: NC, 9: NC, 10: LD Anode (+), 11: LD Cathode (−), 12: NC, 13: NC, 14: TEC (−).

14-pin butterfly lasers can be directly clamped to the LD quick-release socket. Additionally, the authors recommend mounting the laser package to a heatsink to dissipate excess heat. This board is capable of driving Type 1 butterfly lasers with the following pin assignments (see Fig. 4):

The case itself is connected to ground, which is indicated by a notional pin 15 in the schematics. For certain rare applications, the user might prefer an external feedback PD for CP operation. In this case, the PD needs to be connected to EXT_PD, and the traces between the solder jumpers JP1, JP2, and JP3 need to be cut.

### Laser Diode Driver

**Fig. 5:**
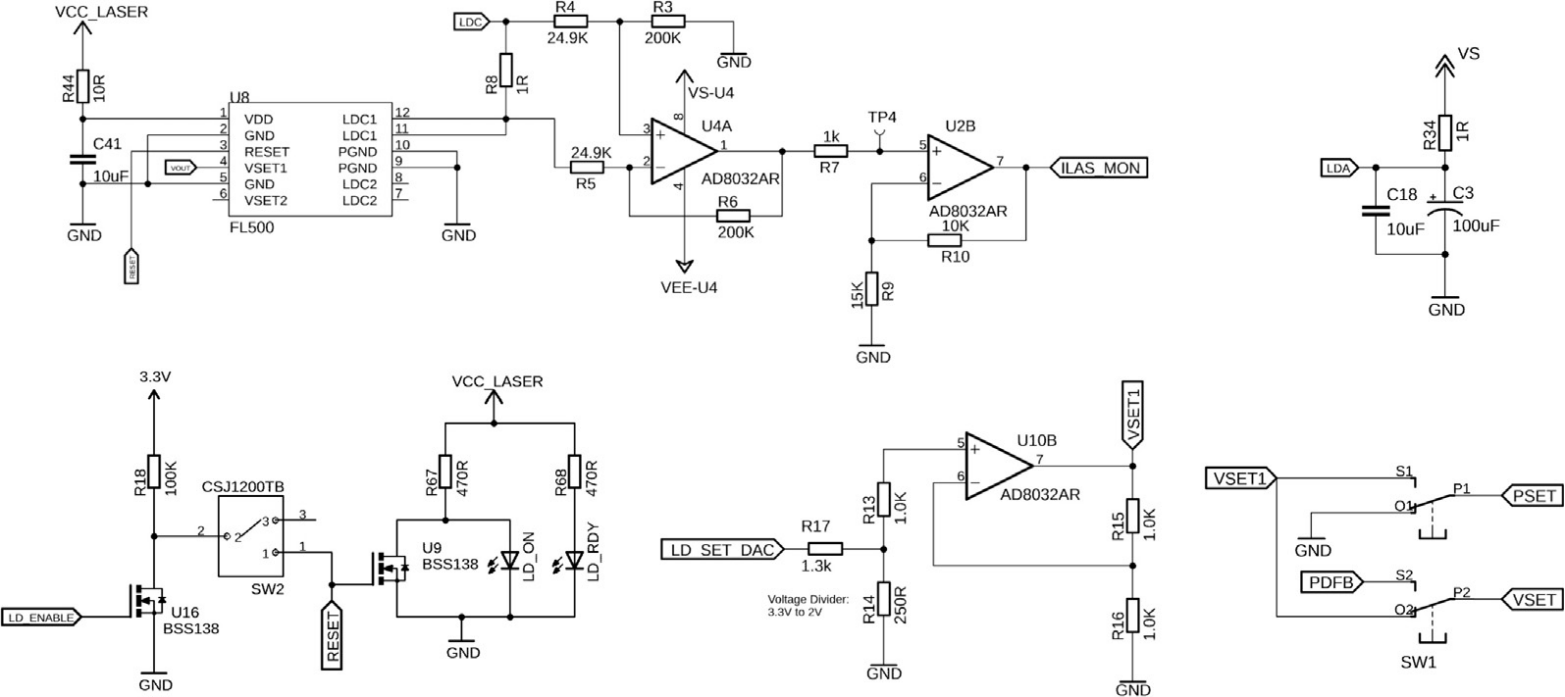
Switch SW1 selects between CC and CP mode. With SW2, the laser can either be disabled manually (e.g., for safety reasons), or controlled via the microcontroller. FL500 (U8) is the laser driver that controls the output laser diode current up to 250 mA by adjusting the voltage out of the LDC1 pin. This voltage output of the FL500 is compared across a 1 Ω resistor with U4A op-amp configuration. The output at TP4 will give a ratio of 250 mA/2V as a one-to-one comparison to the current flowing through the laser diode. U2B further amplifies this value from 2 to 3.3 to utilize the full range of the Teensy ADC. U10B provides signal conditioning for the laser setpoint. U10B can control both CP and CC setpoint, depending on the position of SW1.

The LD is controlled by an FL500 laser driver chip (U8, Wavelength Electronics, Inc., MT, USA). For the laser to operate, it first needs to be enabled. For safety reasons, the LD can be manually disabled by switch SW2 if the RESET pin is left floating (Fig. 5). If the SW2 is in the opposite direction, the laser still needs to be enabled by the microprocessor’s LD_ENABLE pin. When the laser is ready to be operated, the LD_RDY LED will be illuminated, and when it’s actually enabled, the LD_ON LED will turn on as well.

**Fig. 6:**
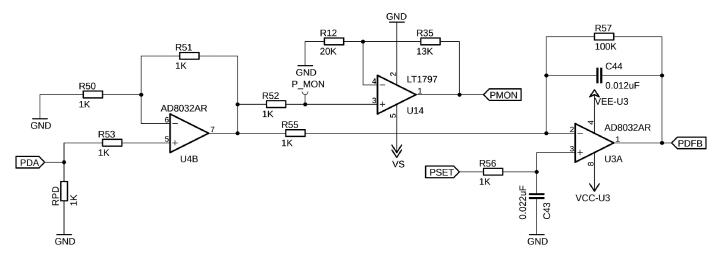
The PDA input current can be adjusted accordingly by changing RPD. This small voltage is amplified by a gain of 2 in U4B and fed into U3A where it is compared to the set PD current and filtered.

As previously mentioned, the laser can be operated in either CC or CP mode, which can be selected by the double-pole, double-throw (DPDT) switch SW1. The respective positions are labeled directly on the PCB. In CC mode, SW1 connects PSET to ground and the analog output of the microcontroller, via some signal conditioning, to the VSET1 input of the FL500 driver. For CP operation, SW1 can be switched in its opposite position, thereby connecting an external PD feedback (PDFB) circuit, which modulates the VSET voltage based on the PD feedback. The maximum photodiode current input for the FL500 is 1 mA. This range can be adjusted with R_PD_, which is set to 1 kΩ in this default design (Fig. 6). This resistor can be replaced based on the expected maximum photodiode current with the following equation^1^:

$$R_{\mathrm{PD}}=\frac{1}{I_{\mathrm{PMON}}}$$

With R_PD_ in Ω. The following equation can be used to convert the measured PMON voltage to photodiode current:

$$I_{\mathrm{PMON}}=\frac{V_{\mathrm{PMON}}}{2R_{\mathrm{PD}}}$$

With I_PMON_ in A.

**Fig. 7:**
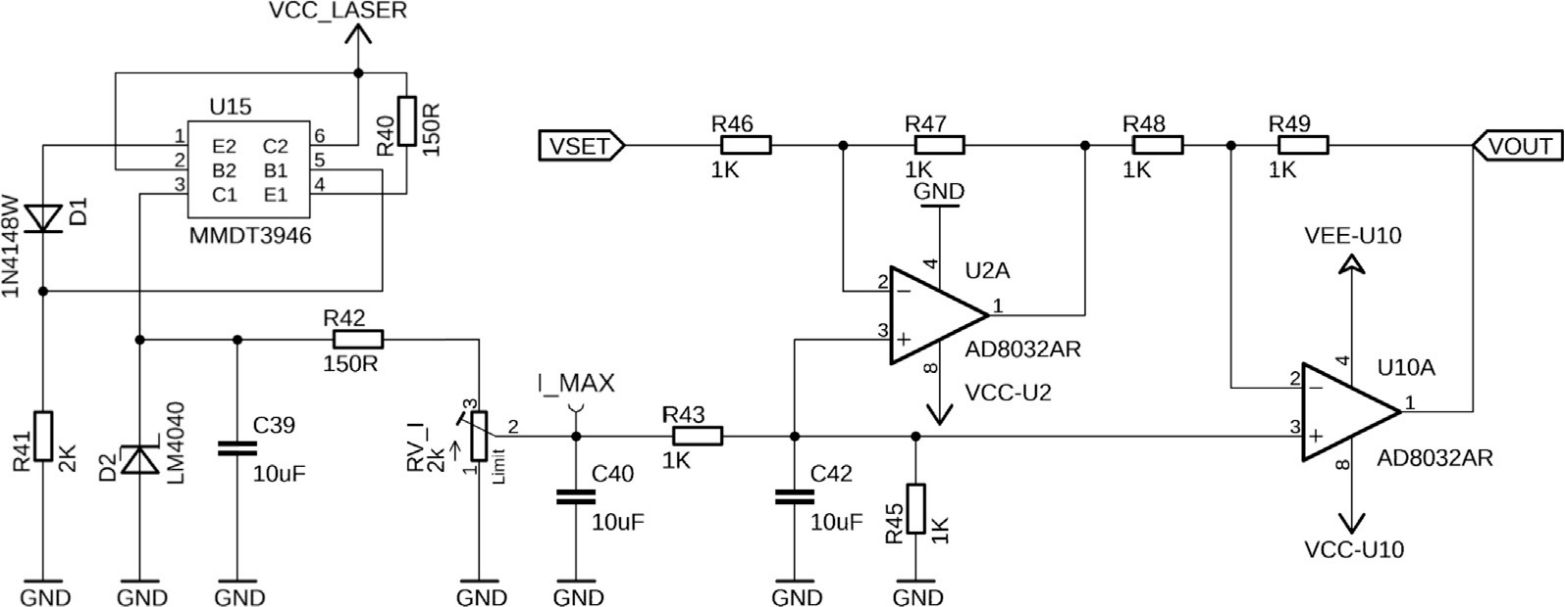
This circuitry allows the operator to adjust the maximum current that flows through the laser diode. The FL500 is capable of delivering up to 250 mA in single-mode operation. Thus, by adjusting the RV_I potentiometer, the VSET input will be attenuated to the value measured at I_MAX at the output VOUT.

In either mode, the PD current can be monitored by the microcontroller’s analog-to-digital converters (ADC) via PMON, and the LD current via ILAS_MON. To protect the laser from over-current, a current limit can be set by the potentiometer RV_I (Fig. 7). If the limit is exceeded, the power VCC_LASER to the laser driver U8 is cut by the NPN-PNP transistor U15. The set limit can be measured at test point I_MAX, and can be calculated by the transfer function of 0.125 A/V. To get the value in A, multiply I_MAX voltage by 0.125. It is recommended to set I_MAX to 0 first and slowly increase the potentiometer to the desired current.

### Laser Temperature Controller

Many laser diodes require active temperature control for precise power and wavelength stability. This board is equipped with a MOT3000-25 (U5, Modular One Technology LLC, TX, USA) precision temperature controller for Peltier modules. This driver’s internal loop compensation is optimized for laser module assemblies such as 14-pin butterfly packages, provides a maximum output current of ±2.5 A, and temperature stability of ±0.002 °C.

**Fig. 8:**
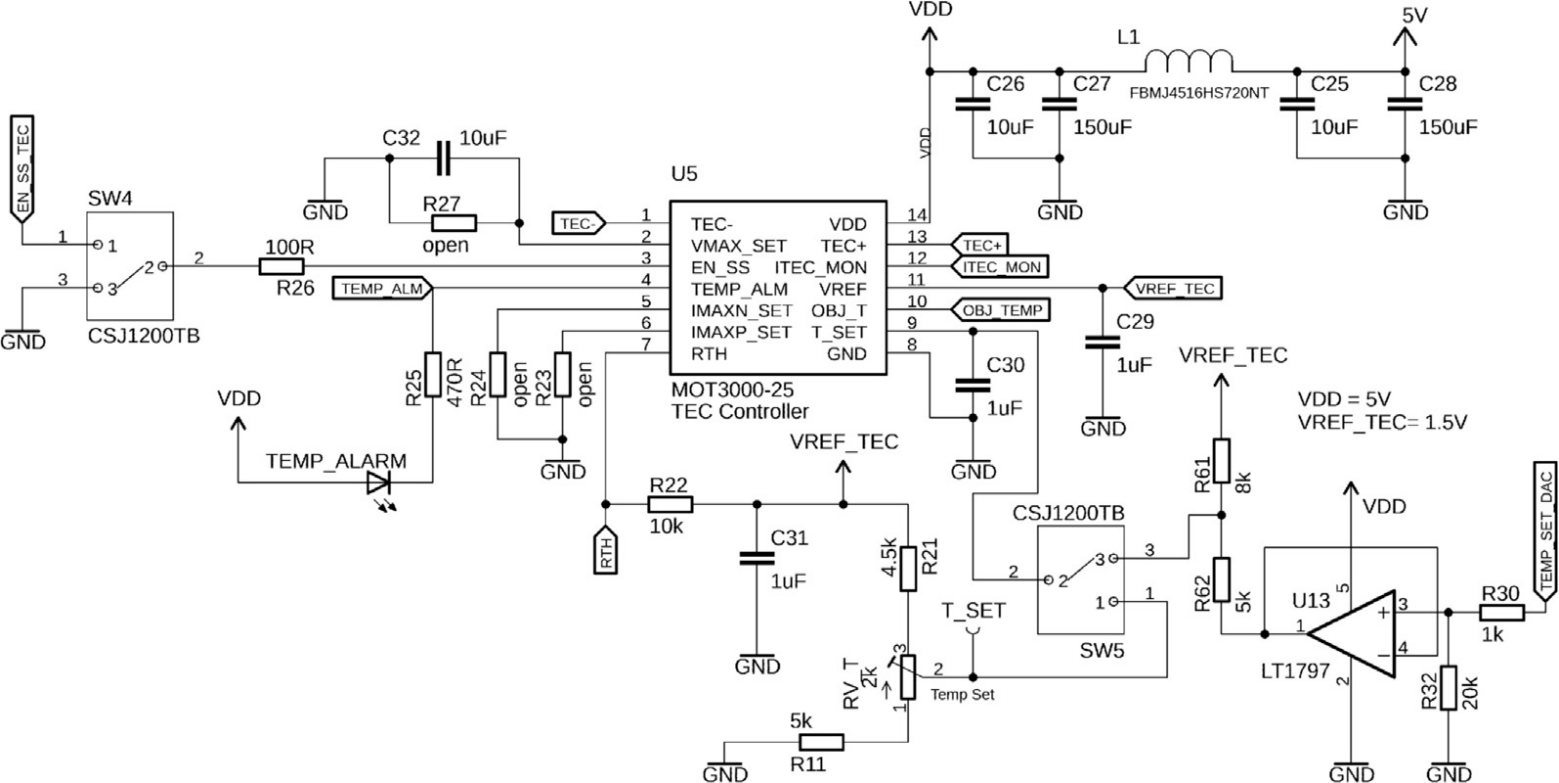
Switch SW 4 allows for manual disabling of the MOT3000-25 TEC controller (U5), or for toggling on/off by the microcontroller. Also, the temperature setpoint can either be controlled manually by the variable resistor RV_T, or through an analog signal from the Teensy, depending on the position of SW5. If the laser temperature is outside a ±1.5 °C window around the set temperature, the TEMP_ALARM LED will illuminate. If desired, the maximum output current (IMAX) and the maximum output voltage (VMAX) of the TEC controller can be limited by soldering resistors at positions R23, R24, and R27, respectively. The reader is referred to the MOT3000-25 datasheet for the appropriate values.

The TEC controller can be disabled by switch SW4, if the EN_SS line is pulled to ground (Fig. 8). If the switch is in the opposite direction, the TEC can be enabled remotely by the microcontroller. The laser temperature can either be set manually (“set-and-forget”) by the potentiometer RV_T while monitoring test point T_SET, or by setting it via the microcontroller. Switch SW5 lets the user choose between the two options. To convert the set voltage into temperature, the user is referred to the MOT3000-25 datasheet, as well as on how to limit the maximum TEC current and voltage by choosing appropriate resistors for R23, R24, and R27 (Fig. 8). By default, these resistor positions can be left open-circuited. If the laser temperature moves outside a ±1.5 °C window around the set temperature, the TEMP_ALARM LED will illuminate and also inform the microcontroller by pulling the line TEMP_ALM low. During operation, the object temperature, as well as the TEC current, can be read in real-time by the microcontroller’s ADCs via the analog signals OBJ_TEMP and ITEC_MON.

**Fig. 9:**
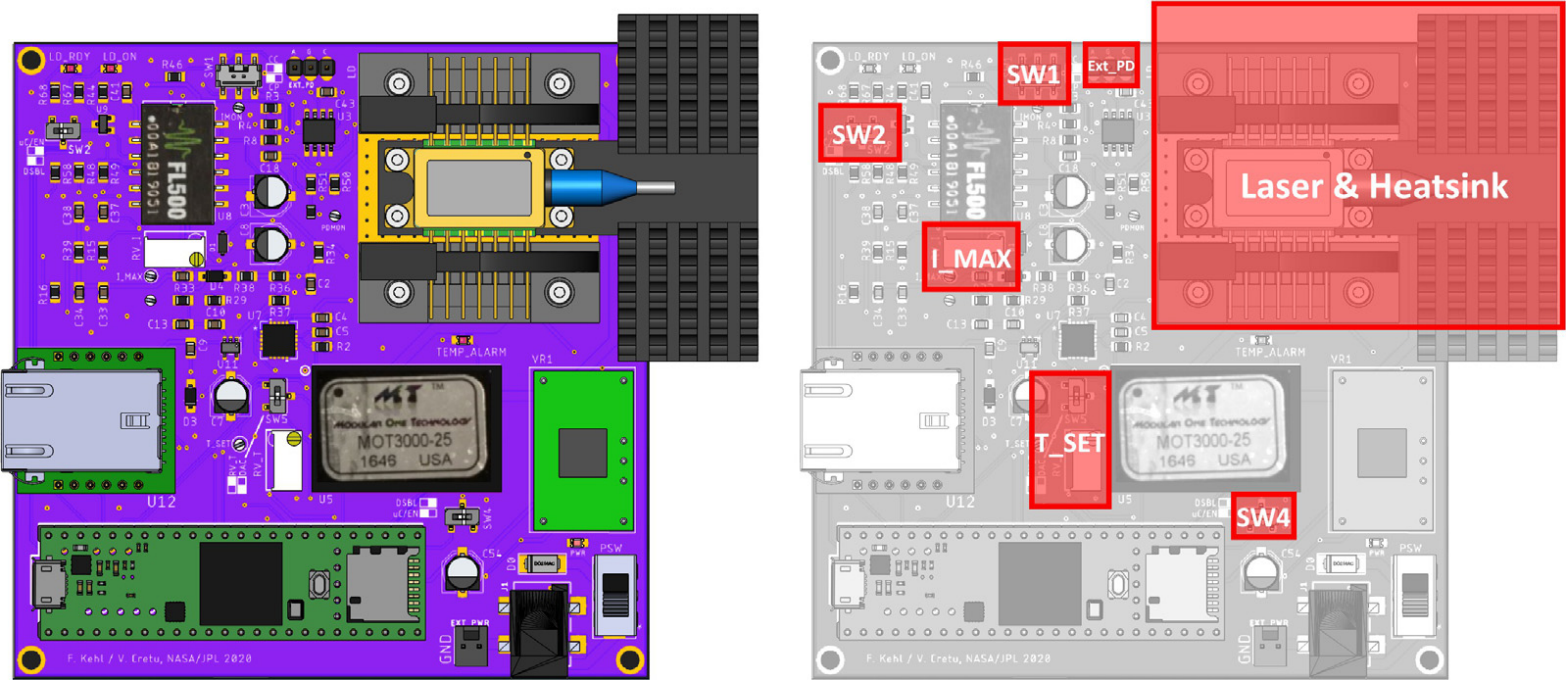
CAD top view of the assembled board (left), together with a map of the board, highlighting critical hardware elements, such as the laser and the heatsink, switches SW1, SW2, SW4, where the external PD can be connected, and where the maximum laser current (I_MAX) can be set, as well as the TEC temperature (T_SET).

**Fig. 10:**
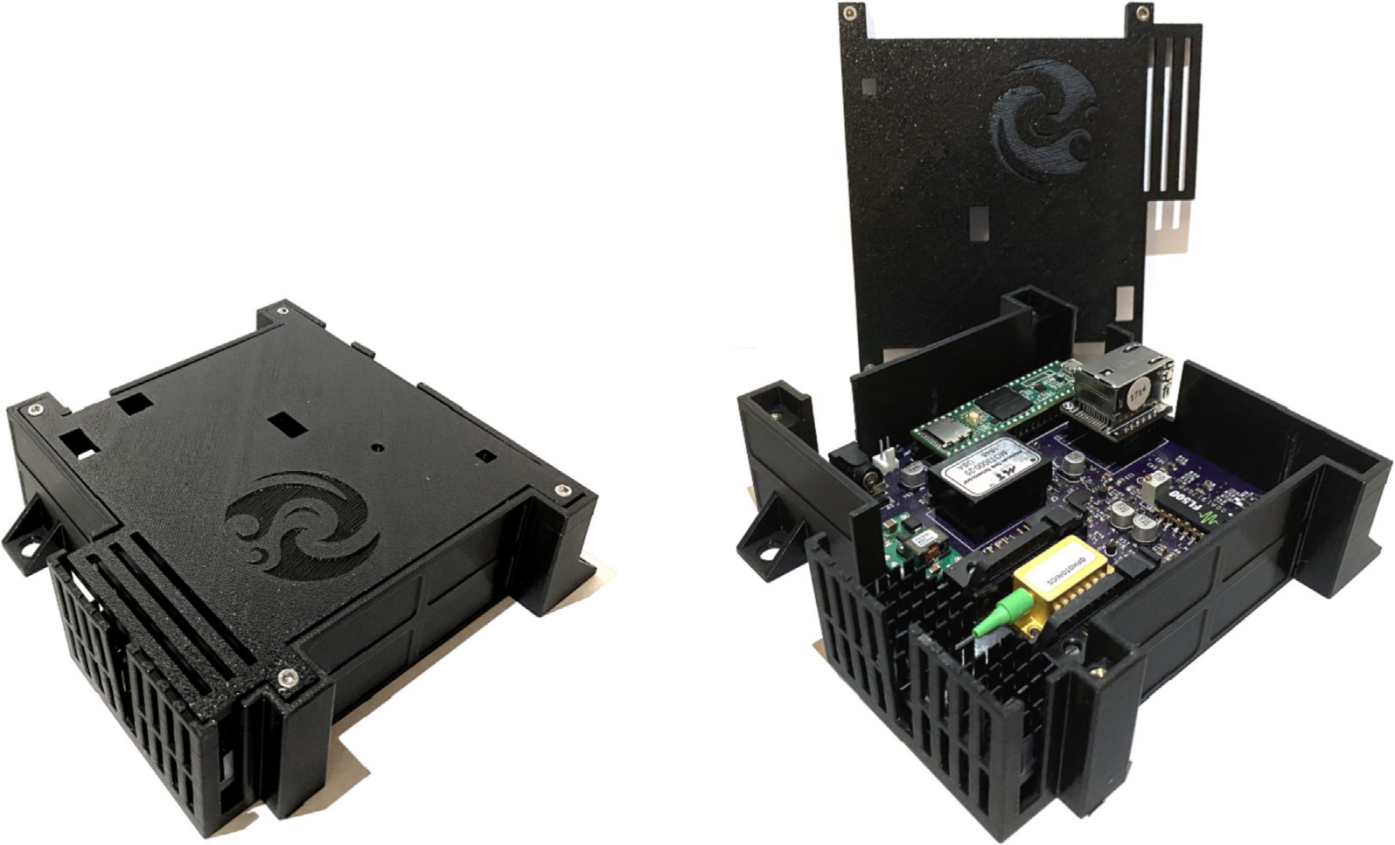
Laser board mounted in 3D-printed enclosure.

Fig. 9 provides an overview of the different functional elements and where they are located on the board. A CAD model of the assembled board, as well as the PCB design files, are provided with the supplementary material. If desired, a CAD file for a 3D printable, custom enclosure for the laser board is provided with this manuscript, comprising a bottom part and a lid (Fig. 10).

## Design Files

*Design* F*ilesSummary*

**Table:** 

**Design file name**	**File type**	**Open source license**	**Location of the file**
Laser PCB V1.0.sch	Schematics	*CC BY 4.0*	*https://osf.io/rydxk/*
Laser PCB V1.0.brd	Layout	*CC BY 4.0*	*https://osf.io/qhsz4/*
Laser PCB V1.0.lbr	Library	*CC BY 4.0*	*https://osf.io/ur95c/*
Laser_Board_Firmware_V1.0.zip	Firmware	*CC BY 4.0*	*https://osf.io/dnztr/*
Laser PCBA Parasolid.x_t	CAD	*CC BY 4.0*	*https://osf.io/f4qk2/*
Laser Enclosure Box.STL	CAD, 3D-printing	*CC BY 4.0*	*https://osf.io/9vp65/*
Laser Enclosure Lid.STL	CAD, 3D-printing	*CC BY 4.0*	*https://osf.io/yfr2p/*

**Table:** 

**File**	**Description**
Laser PCB V1.0.sch	Autodesk Eagle schematic file. The PCB schematic can be viewed and edited if desired.
Laser PCB V1.0.brd	Autodesk Eagle board file. Required to produce the PCB.
Laser PCB V1.0.lbr	Autodesk Eagle part library for the PCB.
Laser_Board_Firmware_V1.0.zip	Contains the *.ino, *.cpp, and *.h files to program the Teensy controller.
Laser PCBA Parasolid.x_t	3D model of the PCB assembly.
Laser Enclosure Box.STL	STL-File to 3D-print the enclosure box.
Laser Enclosure Lid.STL	STL-File to 3D-print the enclosure lid.

## Bill of Materials

***PCB assembly (PCBA):***

**Table:** 

**Designator**	**Component**	**Number**	**Cost per unit - USD**	**Total cost -USD**	**Supplier**	**Supplier Part. No.**	**Manufacturer Part. No.**
C1, C6, C35, C36, C45, C46, C47, C50, C51, C52, C53	CAP CER 0.1UF 50 V X7R 0805	11	0.07	0.80	Digi-Key	478-10836-1-ND	08055C104KAT4A
C10	CAP CER 1200PF 50 V X7R 0805	1	0.11	0.11	Digi-Key	399-8018-1-ND	C0805C122K5RACTU
C13, C23, C24, C37, C38	CAP CER 22UF 25 V X5R 0805	5	0.47	2.35	Digi-Key	490-10749-1-ND	GRM21BR61E226ME44L
C2	CAP CER 47UF 10 V X5R 0805	1	0.77	0.77	Digi-Key	490-9961-1-ND	GRM21BR61A476ME15L
C21, C33	CAP CER 0.47UF 25 V X7R 0805	2	0.1	0.2	Digi-Key	1276-6480-1-ND	CL21B474KAFNNNG
C22	CAP CER 4.7UF 25 V X5R 0805	1	0.11	0.11	Digi-Key	1276-1244-1-ND	CL21A475KAQNNNE
C27, C28	CAP CER 150UF 6.3 V X5R 1206	2	1.14	2.28	Digi-Key	490-13969-1-ND	GRM31CR60J157ME11L
C3, C7, C8, C54	CAP ALUM 100UF 20% 16 V SMD	4	0.43	1.72	Digi-Key	P15086CT-ND	EEE-FT1C101AR
C4, C12, C14, C18, C20, C25, C26, C32, C34, C39, C40, C41, C42, C55, C56	CAP CER 10UF 16 V X5R 0805	15	0.08	1.25	Digi-Key	1276-6455-1-ND	CL21A106KOQNNNG
C43	CAP CER 0.022UF 16 V X7R 0805	1	0.1	0.1	Digi-Key	732-8041-1-ND	885,012,207,041
C44	CAP CER 0.012UF 50 V X7R 0805	1	0.13	0.13	Digi-Key	399-9171-1-ND	C0805C123K5RACTU
C5, C9, C29, C30, C31	CAP CER 1UF 25 V X7R 0805	5	0.1	0.5	Digi-Key	1276-1066-1-ND	CL21B105KAFNNNE
D0	DIODE SCHOTTKY 40 V 2A DO214AC	1	0.46	0.46	Digi-Key	641-1698-1-ND	CDBA240LL-HF
D1	DIODE GEN PURP 100 V 150MA SOD123	1	0.17	0.17	Digi-Key	1N4148WRHGCT-ND	1N4148W RHG
D2	IC VREF SHUNT 0.5% SOT23	1	0.64	0.64	Digi-Key	LM4040C25QFTADICT-ND	LM4040C25QFTA
D3, D4	DIODE SCHOTTKY 20 V 500MA SOD123	2	0.29	0.58	Digi-Key	MBR0520LT1GOSCT-ND	MBR0520LT1G
D5	DIODE SCHOTTKY 40 V 1A SMB	1	0.37	0.37	Digi-Key	MBRS140T3GOSCT-ND	MBRS140T3G
EXT_PWR	CONN HEADER VERT 2POS 2.54MM	1	0.11	0.11	Digi-Key	A1921-ND	640456-2
L1	FERRITE BEAD 72 OHM 1806 1LN	1	0.19	0.19	Digi-Key	587-1776-1-ND	FBMJ4516HS720NT
L2, L3	FIXED IND 47UH 620MA 600MOHM SMD	2	0.76	1.52	Digi-Key	SRR4018-470YCT-ND	SRR4018-470Y
LD_ON	LED BLUE CLEAR 0805 SMD	1	0.18	0.18	Digi-Key	732-4982-1-ND	150080BS75000
LD_RDY	LED YELLOW CLEAR 0805 SMD	1	0.18	0.18	Digi-Key	732-4987-1-ND	150080YS75000
P_MON, TP1, TP3, TP4, T_SET	PC TEST POINT COMPACT BLACK	5	0.4	2	Digi-Key	36-5006-ND	5006
PSW	SWITCH SLIDE DPDT 300MA 6 V	1	0.55	0.55	Digi-Key	401-2002-1-ND	JS202011SCQN
PWR_LED	LED GREEN CLEAR 0805 SMD	1	0.18	0.18	Digi-Key	732-4986-1-ND	150080VS75000
R1, R10, R20, R22, R29, R31	RES SMD 10 K OHM 1% 1/3W 0805	6	0.1	0.6	Digi-Key	A126417CT-ND	CRGH0805F10K
R12, R19, R32	RES 20 K OHM 1% 1/4W 0805	3	0.1	0.3	Digi-Key	RNCP0805FTD20K0CT-ND	RNCP0805FTD20K0
R14	RES SMD 249 OHM 1% 1/8W 0805	1	0.1	0.1	Digi-Key	13-RT0805FRE07249RLCT-ND	RT0805FRE07249RL
R17	RES SMD 1.3 K OHM 5% 1/2W 0805	1	0.1	0.1	Digi-Key	P1.3KADCT-ND	ERJ-P06J132V
R18, R57	RES 100 K OHM 1% 1/8W 0805	2	0.1	0.2	Digi-Key	RMCF0805FT100KCT-ND	RMCF0805FT100K
R2, R11, R62	RES SMD 5 K OHM 1% 1/8W 0805	3	0.13	0.39	Digi-Key	541-4321-1-ND	CRCW08055K00FKTA
R21	RES SMD 4.53KOHM 0.5% 1/10 W 0805	1	0.1	0.1	Digi-Key	RR12P4.53KDCT-ND	RR1220P-4531-D-M
R25, R67, R68, R75	RES 470 OHM 1% 1/8W 0805	4	0.1	0.4	Digi-Key	RMCF0805FT470RCT-ND	RMCF0805FT470R
R26, R33	RES 100 OHM 1% 1/4W 0805	2	0.1	0.2	Digi-Key	RNCP0805FTD100RCT-ND	RNCP0805FTD100R
R28	RES SMD 15.8 K OHM 1% 1/8W 0805	1	0.1	0.1	Digi-Key	311-15.8KCRCT-ND	RC0805FR-0715K8L
R3, R6	RES 200 K OHM 5% 1/8W 0805	2	0.1	0.2	Digi-Key	RMCF0805JT200KCT-ND	RMCF0805JT200K
R35	RES 13 K OHM 1% 1/8W 0805	1	0.1	0.1	Digi-Key	RMCF0805FT13K0CT-ND	RMCF0805FT13K0
R36, R37, R44, R58, R59, R60, R63, R64, R65, R66	RES SMD 10 OHM 1% 1/3W 0805	10	0.09	0.89	Digi-Key	A126420CT-ND	CRGH0805F10R
R4, R5	RES SMD 25.5 OHM 1% 1/8W 0805	2	0.1	0.2	Digi-Key	541-25.5CCT-ND	CRCW080525R5FKEA
R40, R42	RES 150 OHM 1% 1/4W 0805	2	0.1	0.2	Digi-Key	RNCP0805FTD150RCT-ND	RNCP0805FTD150R
R41	RES 2 K OHM 1% 1/4W 0805	1	0.1	0.1	Digi-Key	RNCP0805FTD2K00CT-ND	RNCP0805FTD2K00
R61	RES 8.06 K OHM 1% 1/8W 0805	1	0.1	0.1	Digi-Key	RMCF0805FT8K06CT-ND	RMCF0805FT8K06
R7, R13, R15, R16, R30, R43, R45, R46, R47, R48, R49, R50, R51, R52, R53, R55, R56, RPD	RES SMD 1 K OHM 1% 1/3W 0805	18	0.09	1.60	Digi-Key	A126422CT-ND	CRGH0805F1K0
R71	RES 21 K OHM 1% 1/8W 0805	1	0.1	0.1	Digi-Key	738-RMCF0805FT21K0CT-ND	RMCF0805FT21K0
R8, R34, R38, R39	RES 1 OHM 1% 1/4W 0805	4	0.1	0.4	Digi-Key	RNCP0805FTD1R00CT-ND	RNCP0805FTD1R00
R9	RES SMD 15 K OHM 1% 1/8W 0805	1	0.1	0.1	Digi-Key	13-RE0805FRE0715KLCT-ND	RE0805FRE0715KL
RSHUNT1	RES 0.1 OHM 1% 7 W 2512	1	1.3	1.3	Digi-Key	511-1692-1-ND	GMR100HTCFAR100
RV_I, RV_T	TRIMMER 2 K OHM 0.5 W PC PIN TOP	2	1.5	3.0	Digi-Key	490-2880-ND	PV36W202C01B00
SW1	SWITCH SLIDE SPDT 6A 120 V	1	4.17	4.17	Digi-Key	CKN5001-ND	1101M2S3CQE2
SW2, SW4, SW5	SWITCH SLIDE SPDT 100MA 6 V	3	0.79	2.37	Digi-Key	563-1022-1-ND	CJS-1200 TB
TEMP_ALARM	LED RED CLEAR 0805 SMD	1	0.18	0.18	Digi-Key	732-4984-1-ND	150080RS75000
U1	IC CURRENT MONITOR 0.5% SOT23-5	1	2.51	2.51	Digi-Key	296-26063-1-ND	INA169NA/3K
U11	IC REG MULT CONFG ADJ SOT23-5	1	5.04	5.04	Digi-Key	LT1611CS5#TRMPBFCT-ND	LT1611CS5#TRMPBF
U13, U14	IC OPAMP GP 1 CIRCUIT TSOT23-5	2	2.54	5.08	Digi-Key	LT1797CS5#TRMPBFCT-ND	LT1797CS5#TRMPBF
U15	TRANS NPN/PNP 40 V 0.2A SOT363	1	0.43	0.43	Digi-Key	MMDT3946-FDICT-ND	MMDT3946-7-F
U2, U3, U4, U10	IC OPAMP VFB 2 CIRCUIT 8SOIC	4	5.35	21.4	Digi-Key	AD8032ARZ-REEL7CT-ND	AD8032ARZ-REEL7
U6	IC TRNSLTR BIDIRECTIONAL 20TSSOP	1	1.29	1.29	Digi-Key	296-21527-1-ND	TXB0108PWR
U7	IC REG LINEAR POS ADJ 1A 20VQFN	1	4.9	4.9	Digi-Key	296-39503-1-ND	TPS7A4700RGWR
U9, U16	MOSFET N-CH 50 V 220MA SOT-23	1	0.26	0.52	Digi-Key	BSS138CT-ND	BSS138
UC	TEENSY 3.5 W/OUT HDRS K64 EVAL	1	26.25	26.25	Digi-Key	1568-1443-ND	DEV-14055
VR1	DC DC CONVERTER 2.5–12.6 V	1	19.99	19.99	Digi-Key	296-20525-ND	PTN78060WAZ
U12	Networking Modules W5200	1	23.91	23.91	Mouser	950-WIZ820IO	WIZ820io
U5	TEC CONTROLLER	1	82.25	82.25	Modular One Technology	MOT3000-25	MOT3000-25
Heatsink	Heat Sink for Butterfly Laser Package	1	38.85	38.85	Modular One Technology	MOT_BT_HS	MOT_BT_HS
J1	DC Barrel Power Jack/Connector (SMD)	1	1.5	1.5	Sparkfun	PRT-12748	PRT-12748
LD Socket	Laser Diode Socket, left	1	28.44	28.44	Azimuth Electronics	5254-100-07S	5254-100-07S
LD Socket	Laser Diode Socket, right	1	28.44	28.44	Azimuth Electronics	5254-100-07R	5254-100-07R
U8	500 mA Laser Diode Driver	1	56	56	Wavelength Electronics	FL500	FL500
PCB	Custom	1	26.03	26.03	OshPark	Custom	Custom
Socket Head Screw for Laser	Socket Head Screw, M2 × 0.4, 6 mm	4	0.12	0.48	McMaster-Carr	91290A01	91290A01
Socket Head Screw for Laser Socket	Socket Head Screw, M2 × 0.4, 10 mm	4	0.07	0.27	McMaster-Carr	91292A833	91292A833
Solderable Nuts	RND STANDOFF M2X0.4 STEEL 1.5MM	8	1.09	8.72	Digi-Key	732-7069-1-ND	9774015243R
Solderable Corner Standoffs	ROUND STANDOFF M2X0.4 STEEL 8MM	4	0.95	3.8	Digi-Key	732-7111-1-ND	9774080243R

***Enclosure:***

**Table:** 

**Designator**	**Component**	**Number**	**Cost per unit - USD**	**Total cost - USD**	**Source: McMaster-Carr part number**	**Material type**
Heat Inserts	4–40 Tapered Heat-Set Inserts for Plastic	8	0.1265	1.012	93365A122	Brass
Screws	18–8 Stainless Steel Socket Head Screws	8	0.0481	0.3848	92196A105	Stainless Steel

## Build Instructions

***PCBA:***

**Fig. 11:**
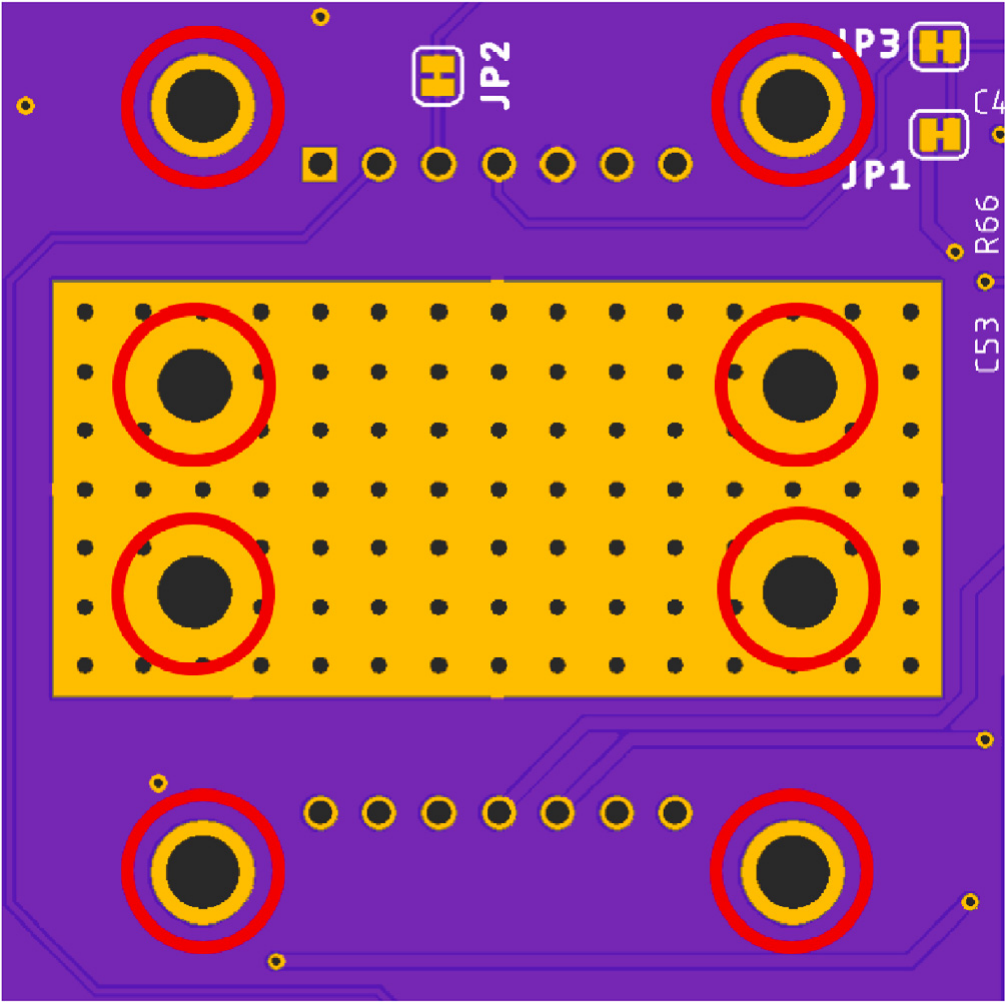
Solder nuts to the eight through-holes (red circles) to mount the laser (inner four holes) and the laser socket (outer four holes) using M2 screws. (For interpretation of the references to colour in this figure legend, the reader is referred to the web version of this article.)

• Solder all SMD components to the PCB first, followed by soldering all through-hole components and connectors.
• Solder the eight “Solderable Nuts” to the annular ring of the through-holes under the laser socket, on the bottom side of the PCB (Fig. 11). The four inner nuts allow for securing the laser. The four outer holes are for the laser socket.

The Corner Standoffs can be soldered to the four corner through-holes if the board is used without the provided 3D-printed enclosure. If the board is mounted to the enclosure, these standoffs are not required.

**Fig. 12:**
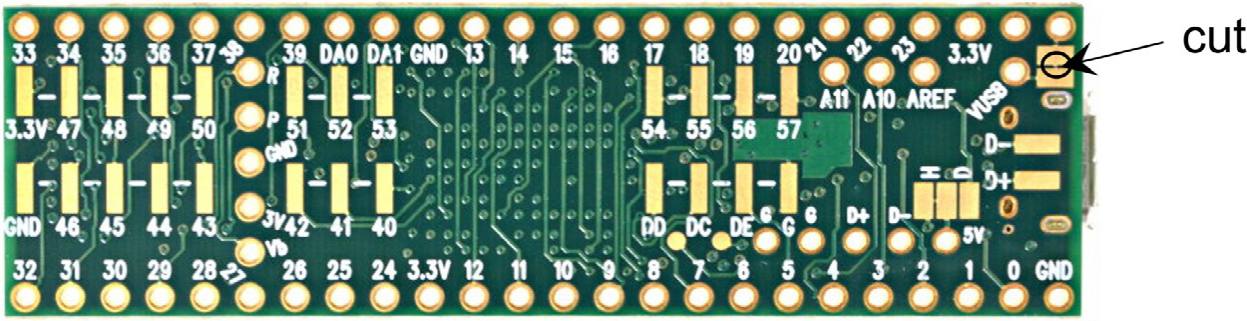
Bottom side of a Teensy 3.5. For nominal operation with the presented board, the indicated trace needs to be cut.
• Although the Teensy controller itself and other components such as the TEC controller or the Ethernet module could be directly soldered to the mainboard, the authors recommend using female socket connectors for easy replacement. Make sure to mount these components in the right orientation.
• The network module (U12) is an optional feature and does not have to be installed if no Ethernet interface is required.
• If this board is used for the first time, it is recommended to skip this step first and go directly to Section *Operation Instructions*, and then return here before installing the laser. The laser can directly be mounted together with the heatsink by clamping it with the socket’s lids. Lasers can be particularly ESD-sensitive, and it is therefore recommended to wear a grounding strap when handling the laser. It is also recommended to apply a small amount of thermal paste between the laser and the heatsink for better heat transfer. Align the holes of the laser, the heatsink, and the PCB and use four M2 screws to secure the assembly to the board.
• Lasers are potentially hazardous and can cause damage to eyes and/or burns to skin. The operation of lasers should only be carried out by trained professionals wearing appropriate personal protective equipment and in a controlled environment.

• Cut the trace between the two solder jumpers at the top right on the back of the Teensy to separate VIN from VUSB, to use the power provided by the mainboard (Fig. 12).

## *Enclosure:*

• 3D print enclosure bottom and lid.
• Install 4–40 (or M3) heat inserts or tap screw holes directly.
• Mount laser board inside the bottom enclosure using four 4–40 (or M3) screws.
• Close lid using four 4–40 (or M3) screws.

## Operation Instructions

### Prepare Hardware for First Operation

It is recommended that for these steps, no laser is installed to avoid any damage due to unknown initial settings.

1. Select with switch SW1 whether the laser shall be operated in CC or CP mode.
2. Enable the TEC with switch SW4 and set it to manual temperature control with switch SW5.
3. Connect the external power supply to J1 and switch the main power on.
4. To avoid thermal damage to the laser: measure the voltage at test point T_SET by turning the variable resistor RV_T, and adjusting it to 0.75 V, which corresponds to a default, hardware-set laser temperature of 25 °C. If a different temperature is required, the user is referred to the MOT3000-25 datasheet.
5. If the temperature needs to be adjusted by the microcontroller, SW4 can now safely be switched.
6. To avoid accidental over-current operation of the laser: identify the maximum forward current of the designated laser from its datasheet. Measure the voltage at test point I_MAX while adjusting the variable resistor RV_I to set it to the desired current limit. The voltage measured at I_MAX has a ratio of 0.125 A/V. It is recommended that RV_I is turned so that I_MAX TP measures 0 V. Then, once the laser or dummy load is installed, slowly adjust RV_I by measuring TP4 voltage. Please note that the transfer function at TP4 is 250 mA/V. This will give an accurate reading of the laser current limit and protect the laser diode.
7. At this point, it’s safe to install the laser as described in Section *Build Instructions*.

### Upload Firmware

In this section, the initial programming of the microcontroller is explained. Unless the user wishes to customize the firmware, this only needs to be done once. If the Teensy controller needs to be replaced, repeat steps 4–6 below.

1. Download and install Arduino IDE (or similar).
2. Download and install Teensyduino.^2^
3. Download *.ino file from document repository and open in the Arduino IDE, under “File > Open…”.
4. Connect the power supply and USB cable. Make sure the main power switch on the board is in the ON position.
5. In the Arduino IDE, go to “Tools > Board” and select Teensy 3.5, and under “Tools > Port” the corresponding COM-port where your Teensy is connected.
6. Go to “Sketch > Upload” to compile the code and program your Teensy controller.

### Direct Control

1. Connect the power supply and USB cable. Make sure the main power switch on the board is in the ON position.
2. Connect the peripherals, such as valves, pumps, and sensors.
3. Establish communication with the board:
  • In Arduino, under “Tools > Port”, select the corresponding COM-port where your Teensy is connected.
  • In Arduino, open Serial Monitor under “Tools > Serial Monitor”.
  • Make sure the Baud Rate is 115,200 and both, NL and CR, are selected.
  • Type individual commands (as specified in the Serial Command Document) in the command line and send by clicking “send” or by pressing “Enter” on the keyboard.
  • Continue by sending the next desired command.

### Serial Command List

Below, a list of serial commands that can be sent to the microcontroller. Most commands must begin with { and end with }. Inside the brackets is the command itself. A command consists of a series of identifying characters and an argument separated by spaces. An example command would be { A B 100 }, where *A* and *B* are the identifying characters, and *100* the argument. It will be listed in the following format:

**Table:** 

**Serial Command**	**Description**
{ A B xxx }	Example command, where e.g., xxx = [0–100].
hello *or* hi	Communication check: *Hello* response.
{ i }	Reads and prints the total current in mA
{ O E x }	Arm/disarm laser (enable/disable Vs). x = [1/0] (ON/OFF)
{ O L E x }	Turn laser on/off. x = [1/0] (ON/OFF) (only works if laser is armed)
{ O L I xxx }	Set laser diode current/power. xxx = [0–255] (8-bit integer)
{ O T E x }	Enable/disable TEC. x = [1/0] (ON/OFF)
{ O T T xx }	Set laser temperature. xx = [15–30] (in °C)
{ O }	Prints telemetry: LD status LD current PD current TEC Temp

**Table:** 

## Validation and Characterization

A single-mode fiber-coupled 488 nm laser diode (QFLD-488-20S, QPhotonics, MI, USA) with a high, typical forward voltage of 6.1 V and maximum optical output of 20 mW was chosen to validate the board’s performance. The laser was operated both in CC and in CP mode over an extended period of time, while the current, power, temperature, and wavelength stability were recorded. The current and temperature were measured using the internal feedback and recorded via the microcontroller. The optical power for CP mode was measured with an S120VC photodiode power sensor in combination with an S120-FC FC/PC fiber adapter and recorded with a PM100USB power meter (all Thorlabs, NJ, USA). The wavelength stability was assessed using an Ocean Optics HR4000 spectrometer (Ocean Insight Inc., FL, USA).

**Fig. 13:**
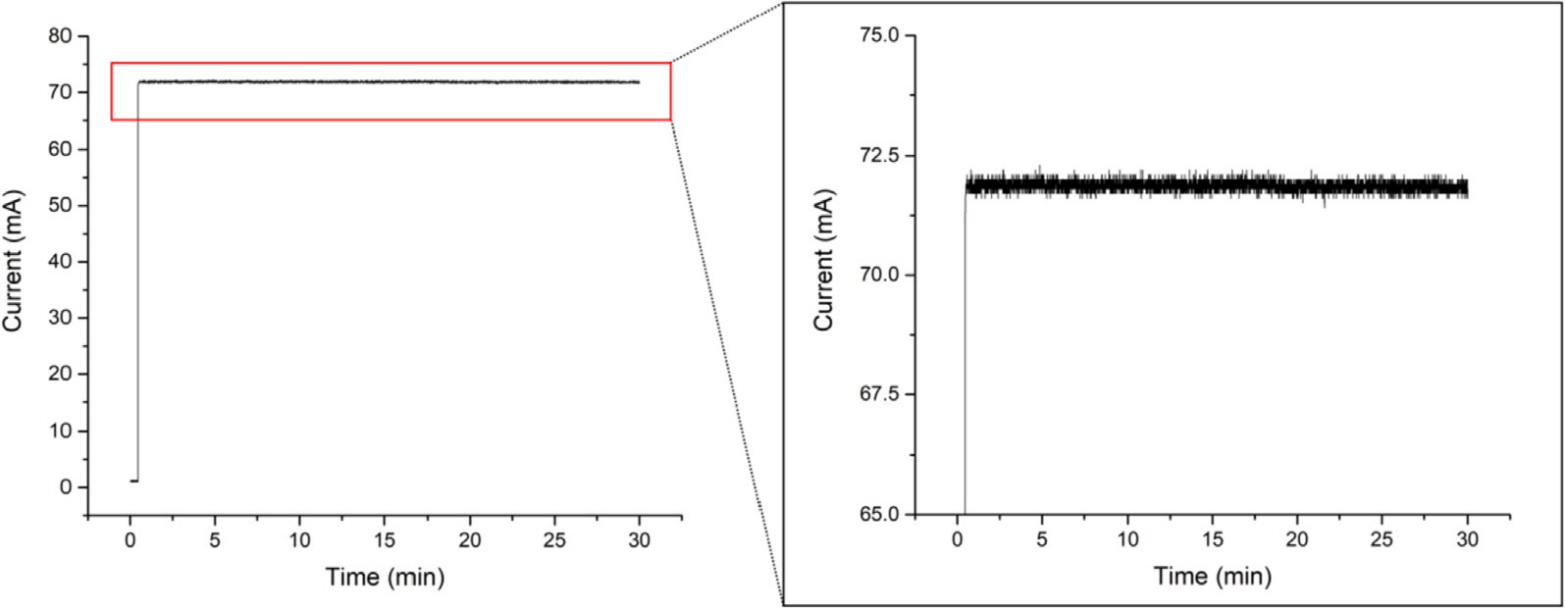
Laser diode current in CC mode over 30 min.

The laser was turned on in CC mode at 0.5 min (Fig. 13). The current of the laser diode immediately stabilized after powering up and was recorded at 5 Hz in CC mode over 30 min. The laser current was set to 70 mA by the Teensy controller, and the resulting forward current averaged at 71.87 ± 0.10 mA. Drift over the duration of the measurement was barely measurable and was on the order of −0.001 mA/min.

**Fig. 14:**
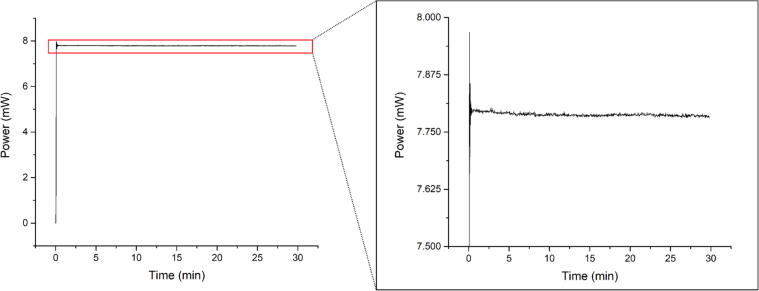
Optical laser output power in CP mode over 30 min.

In CP mode, the laser’s output fiber was connected to the external power meter, and the optical power was recorded at 1 Hz over a duration of 30 min (Fig. 14). The power was set with the microcontroller to a value corresponding to 8 mW. It then settled within a few seconds and then averaged at 7.788 ± 0.004 mW, with a drift of −0.0004 mW/min.

**Fig. 15:**
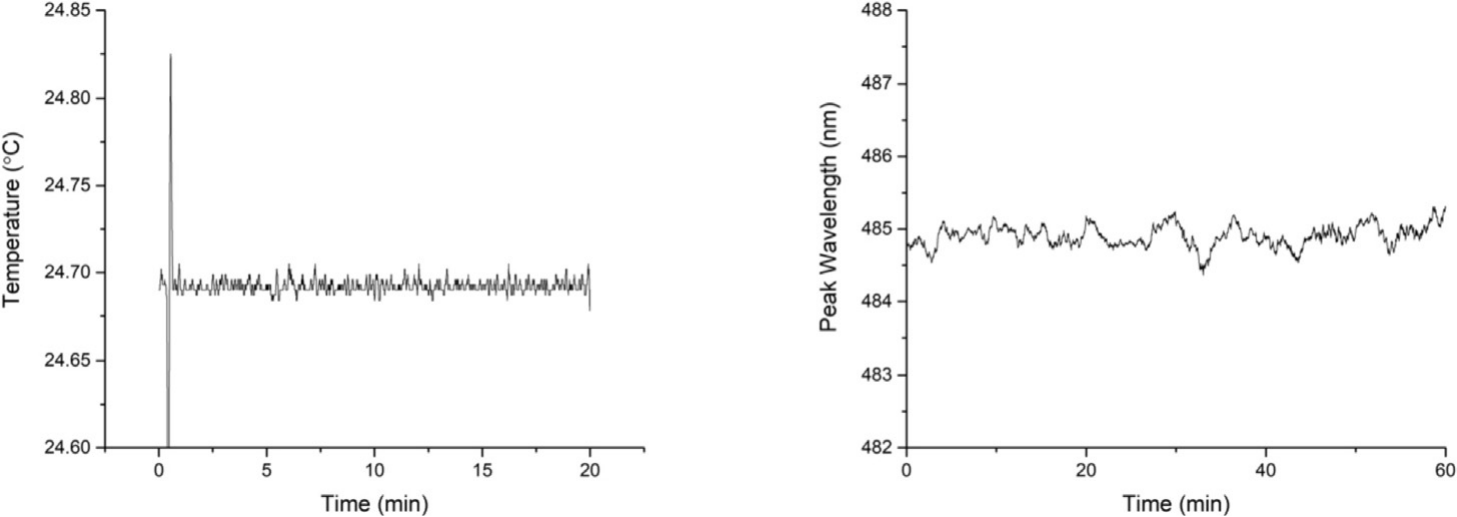
Laser temperature stability over 20 min (left), and peak wavelength stability over 60 min (right).

The TEC temperature was set to 25.00 °C, briefly turned off after a few seconds to demonstrate settling time, and then recorded over 20 min at 5 Hz (Fig. 15, left). The temperature settled after a few seconds and averaged at 24.69 ± 0.014 °C. While the absolute temperature was off by −0.31 °C (which is within the 5% resistance accuracy of the butterfly’s internal 10 kΩ thermistor element), the temperature was also within the <±0.02 °C as specified by the MOT3000-25 TEC controller. Because it was so marginal, no meaningful temperature drift could be quantified.

The peak emission wavelength of the laser diode was recorded at 1 Hz over a duration of 60 min (Fig. 15, right), with a mean of 484.86 ± 0.76 nm, which is within the laser’s specifications of 484–492 nm. While the emission wavelength depends on the laser dye itself, the stability of <1 nm standard deviation is a good indicator for precise laser power and temperature control.

## Declaration of Competing Interest

The authors declare that they have no known competing financial interests or personal relationships that could have appeared to influence the work reported in this paper.

## Footnotes

1 https://www.teamwavelength.com/download/Datasheets/fl591.pdf.

## Footnotes

2 https://www.pjrc.com/teensy/td_download.html.

## References

[1] Andrews D.L. (2012).

[2] Hering P., Lay J.P., Stry S. (2013).

[3] Jelínková H. (2013).

[4] Dong P., Chen Q. (2017).

[5] Bogue R. (2015).

[6] Venghaus H., Grote N. (2017).

[7] Eichler H.J., Eichler J., Lux O. (2018). Lasers.

[8] Oborny N.J. (2021). A radiation tolerant laser-induced fluorescence detection system for a potential Europa lander mission. Acta Astronaut..

[9] Kehl F. (2021). Open-source lab hardware: a versatile microfluidic control and sensor platform. HardwareX.

[10] Cretu V.F., Kehl F. (2021). Open-source lab hardware: low noise adjustable two-stage gain transimpedance amplifier with DC offset. HardwareX.

